# Maternal Supplementation of *Saccharomyces cerevisiae boulardii* during Late-Gestation through Lactation Differentially Modulated Immune Status and Stress Responsiveness of the Progeny to Farrowing and Weaning Stressors

**DOI:** 10.3390/ani12020164

**Published:** 2022-01-11

**Authors:** Janeen L. Salak-Johnson, Cassidy Reddout, Lily Hernandez, Anne Visconti

**Affiliations:** 1 Department of Animal and Food Sciences, Oklahoma State University, Stillwater, OK 74078, USA; cassidy.reddout@okstate.edu (C.R.); lily.hernandez@okstate.edu (L.H.); 2 Department of Animal Sciences, University of Illinois, Urbana, IL 61801, USA; AVisconti@christensenfarms.com

**Keywords:** chemotaxis, cortisol, lymphocyte proliferation, natural killer cell cytotoxicity, neonates, phagocytosis, stress, weaning, yeast-probiotic

## Abstract

**Simple Summary:**

The present study offers insights into the prenatal and postnatal maternal effects of feeding probiotics to pregnant sows from late-gestation through lactation on progeny immune status and stress responsiveness at birth, suckling, and through 14 days post-weaning. These results provide evidence that the neonate’s immune system and stress responsiveness can be modulated in utero by feeding sows probiotics during gestation. Some immune parameters are also affected through the suckling period. Moreover, the progeny’s immune status and cortisol profiles were differentially affected by weaning stress, and these effects were apparent up to 14-days post-weaning. These results suggest that it is plausible to affect the progeny’s immune status and stress responsiveness in response via feeding the sow probiotics for 60 days.

**Abstract:**

The study aimed to investigate and characterize the maternal effects of feeding *Saccharomyces cerevisiae var. boularddii* (*Scb*) to sows from late-gestation through lactation on progeny cortisol, immune status, and stress responsiveness from birth to 14 days post-weaning. Eighty-four piglets were born to sows fed control (CON) or probiotic (PRO) boluses twice daily for 59 days. Blood samples were obtained at birth and 24 h later to assess prenatal effects; 7, 14, and 21 day-of-age to assess potential developmental effects; and at 24 h, 7, and 14 days post-weaning to assess the effects of weaning stress on immune and cortisol responses. Pigs born to PRO sows had less robust cortisol response and enhanced immune parameters at birth and 24 h later, indicating less stress. In response to weaning, pigs born to and nursed by PRO sows displayed unique cortisol and immune profiles than CON pigs. These results indicate that feeding sows *Scb* probiotics during late gestation reduces stress responsiveness to farrowing stress while increasing immune cell populations. Pigs nursed by PRO sows had a more robust initial cortisol response and enhanced neutrophil function and B-cell lymphocyte proliferation in response to weaning stress. These data imply it may be possible to maternally alter immune and stress responses in utero and during suckling in the short-term and up to 14 days post-weaning. However, more research is needed to optimize this strategy.

## 1. Introduction

In pig production, unavoidable common production practices (i.e., weaning) may suppress or enhance the immune status of the pig, while at other times, stress does not affect immunity. Unfortunately, these unavoidable stressors can exacerbate the disease by compromising the immune system [1]. A study conducted by Clapperton emphasized the need for genetic biomarkers to predict offspring’s resistance to infectious diseases [2]. They found that innate and adaptive immune traits are genetically and phenotypically correlated with average growth rate and performance, regardless of health status. Thus, implying that it is plausible to use innate and adaptive immune biomarkers as metrics for selecting offspring with an increased ability to maintain high-performance levels during periods of unavoidable stress. Establishing a healthy gut in the neonate depends on the environment and maternal factors, especially diet. Piglets are born with a sterile, non-colonized gastrointestinal tract without a functional adaptive immune system [3]; thus, maternal disruption in early colonization events may have profound and long-term effects on the development of the neonate’s mucosal immunity and gut architecture. It has been shown that maternal stress during late-gestation impairs the development and reactivity of the progeny, impacting piglet mortality and disease susceptibility [4]. It has also been speculated that other impingements (stressors) play a crucial role in the host’s capacity to utilize innate and adaptive immune responses that may otherwise compromise animal health and lead to maladaptation resulting in disease outcomes.

Antibiotics are routinely used in healthy production animals to improve feed efficiency and sustain health. However, the therapeutic use of antibiotics in food animals is prohibited and tightly regulated by the European Commission [5,6] due to concerns over emerging antimicrobial resistance in humans and animals related to human health, food safety, and animal well-being. Whereas, until 2017, it was a voluntary phase-out approach in the USA per the Veterinary Feed Directive. Therefore, alternative strategies for controlling food animals’ health and well-being must be developed and optimized. One potential alternative is feeding live microorganisms such as bacteria, yeast, and fungi that possess beneficial health effects for humans and animals called probiotics. *Saccharomyces cerevisiae var. boularddii* (*Scb*) is a non-pathogenic, non-colonizing yeast strain that has been fed to monogastric animals. Studies indicate that feeding *Scb* to pigs reduces the incidence of diarrhea and enhances the growth rate in both pigs and broilers [7,8,9,10]. Moreover, feeding *Scb* to monogastric animals has improved gut health by increasing microbial gut diversity [11] and preventing the secretion of inflammatory cytokines [12]. Others have shown direct effects of feeding probiotic *Scb* to young pigs challenged with lipopolysaccharide (LPS) results in reduced incidence of endotoxin-induced mortality and reduced LPS-induced inflammatory response [13]. At the same time, data are limited on investigating the maternal effects of *Scb* supplementation on prenatal and postnatal immune status and stress responsiveness of the sow’s progeny. Therefore, if the benefits of probiotics are transferable from the dam to piglets during gestation and lactation, we hypothesize that sows fed yeast probiotics, *Scb*, during late-gestation may potentially transfer beneficial immune and stress responsiveness to her progeny *in utero.* Moreover, continuing to feed *sows Scb* through lactation should further improve the health and well-being of the pigs in response to weaning stress. Therefore, the objectives were to assess and characterize the effects of feeding yeast probiotics (*Scb*) to sows during late gestation through lactation on cortisol and immune status of her progeny and stress responsiveness of her piglets to farrowing, litter processing, and weaning stressors.

## 2. Materials and Methods

Under protocol no. IACUC-10037, all animal procedures were approved by the University of Illinois Institutional Animal Care and Use Committee (Urbana, IL, USA).

### 2.1. Animals and Experimental Design

A total of eighty-four female piglets born to parity-2 sows derived from the Genetiporc maternal line kept at the University of Illinois Swine Research Center in Urbana-Champaign (USA) were used in this study. Piglets were selected from 18 L born to sows hand-fed two boluses of either probiotic (PRO; n = 9 sows) or placebo (CON; n = 9 sows) every morning (0600 h) starting at gestational day (GD) 84 through the end of lactation. The probiotic bolus (2 × 10^9^ CFU/g) was composed of monogastric specific yeast known as Levucell SB^®^ (*Saccharomyces cerevisiae* var. *boulardii* (*Scb*) CNCMI-1079; Lallemand Animal Nutrition, Montreal, QC, Canada). The CON bolus was sugar-based and anatomically the same size and shape as the probiotic bolus. All sows were kept inside a mechanically-ventilated gestation house in standard individual gestation stalls (61 × 216 cm). At GD 112, sows were moved to mechanically-ventilated farrowing rooms and kept in individual farrowing crates until the end of lactation.

The piglets used in this study were randomly selected from sows fed either PRO (n = 42) or CON (n = 42) boluses during late gestation until the end of lactation. Piglets were housed with their dams from birth (0 D-of-age) until 21 D-of-age (weaning). Once piglets were weaned, they were moved as littermates to a nursery pen and fed *ad libitum,* with a standard nursery diet formulated to meet or exceed NRC requirements [14]. Each pen contained one nipple waterer. The rooms that housed the piglets were kept on 10 h of light: 14 h of dark, in which lights came on at 0700 h and went off at 1700 h.

At birth (0 D-of-age), the entire litter was collected, and all piglets were dried off and weighed. Before suckling, blood samples were obtained, and then piglets were immediately returned to their dams. Sows were bled at GD 112 (before moving into farrowing crates) and then at 24 h post-farrowing. Piglets were bled at 1 (pre-and post-litter processing procedures), 7, 14, and 21 (weaning) D-of-age. Once blood samples were obtained from the piglets at 21 D-of-age, they were weaned, and blood samples were also taken from the sows. Twenty-four hours after weaning, pigs were bled and then bled at D7 and D14 post-weaning to determine maternal treatment effects on stress responsiveness of piglets to weaning stress. All blood samples (1, 5, or 10 mL) were obtained via jugular venipuncture using vacutainers containing sodium heparin or EDTA (the procedure lasted < 2 min). The pigs were held in a supine position, and sows were nose-snared.

### 2.2. Total White Blood Cell Counts (WBC) and Leukocyte Differentials

Heparin-treated whole blood was used to determine the total white blood cell (WBC) counts and leukocyte differential counts (DIFF). Total WBC counts were made electronically using a Coulter Z1 particle counter (Beckman Coulter, Miami, FL, USA). Ten µL of whole blood was added to 10 mL of Isoflow^®^ (Beckman Coulter, Miami, FL, USA) and then three drops of ZAP-OGLOBIN^®^ (Beckman Coulter, Miami, FL, USA) were added to lyse the red blood cells, and tubes were mixed. The sample cup was placed in the counting chamber to determine the total WBC count. Whole blood smears were made, fixed in methanol, and then stained with a Hema-3 staining system (Fisher Scientific, Houston, TX, USA). Slides were viewed under a light microscope, and 100 cells per slide were visually counted to determine leukocyte differential percentages.

### 2.3. Cell Isolation and Plasma Analysis

Whole blood samples were centrifuged at 700× *g* for 30 min at 4 °C. Plasma was aspirated, transferred to tubes, and stored at −80 °C until further analysis. Buffy coat was diluted with Roswell Park Memorial Institute (RPMI; Gibco, Carlsbad, CA, USA) medium, layered over Histopaque-1077 (density = 1.077g/mL; Sigma, St. Louis, MO, USA) and −1119 (density = 1.119 g/mL; Sigma, St. Louis, MO, USA), and centrifuged at 700× *g* for 30 min at 25 °C. Lymphocytes were removed from the 1077-layer, washed twice in RPMI, resuspended, and counted. Neutrophils were removed from the 1119-layer, washed once, and then red blood cells were lysed from the neutrophil fraction, washed in RPMI, and counted. Cell concentrations were adjusted with RPMI based on the immune assay requirements.

Plasma cortisol and interleukin-12 (IL-12) were analyzed following the manufacturer’s protocols. Commercial radioimmunoassay validated for porcine cortisol was measured. Plasma samples from heparin-treated whole blood were assayed for cortisol using a Coat-A-Count cortisol kit, following the manufacturer’s protocol (Diagnostic Products Corp., Los Angeles, CA, USA). Briefly, 25 µL of sample or standard were added to antibody-coated tubes in duplicate. Radiolabeled (I125) cortisol was added to tubes and incubated for 45 min at 37 °C in a water bath. The liquid phase was decanted, and radioactivity was counted with a gamma counter. A standard curve used was 0, 10, 50, 100, 200, and 500 µg/mL. Intra- and inter-assay coefficient of variation were 7.0 and 16.5%, respectively. The minimal detectable concentration of cortisol using this assay was approximately 2 ng/mL. The Porcine IL-12/IL-23 Quantikine kit was used to measure IL-12 in plasma samples (R & D Systems, Minneapolis, MN, USA). The minimal detectable concentration of IL-12/IL-23 using this kit was, on average, 9.0 pg/mL.

### 2.4. Immune Assays

Neutrophil chemotaxis and phagocytosis assays were performed to assess the functional aspects of neutrophils. Chemotaxis was measured using an assay previously described by Salak et al. [15]. Briefly, isolated neutrophils were used at a 3 × 10^6^ cells/mL cell concentration. Both recombinant human complement-5a (C5a; 10^−7^ M; Sigma Aldrich, St. Louis, MO, USA) and recombinant human interleukin-8 (IL-8; 100 µg/mL; Sigma Aldrich, St. Louis, MO, USA) were used as chemoattractants (chemotaxis; directed migration), and assay medium was used as control (random migration). Following the incubation period, the polycarbonate filter (pore size 5 µm) was fixed and stained using LeukoStat^®^ I and II solution (Fischer Scientific, Houston, TX, USA). A technician without knowledge of treatments counted four fields/well at 100× with a light microscope. Neutrophil phagocytosis was measured using a flow cytometry-based assay previously described by Jolie et al. [16], with minor modifications described by Niekamp et al. [17]. Briefly, fluorescent beads were pre-incubated for 30 min in non-heat-inactivated porcine serum before adding the fluorescent beads to the samples at a 10:1 (beads-to-neutrophils) ratio. Samples were incubated for 45 min in the dark at room temperature. The percentage of engulfment of beads by neutrophils was evaluated using a flow cytometer.

Natural killer cell (NK) cytotoxicity was measured using a commercially available non-radioactive cytotoxicity detection kit (Roche Diagnostics, Indianapolis, IN, USA) as previously described by Sutherland et al. [18] to assess innate immune status. Briefly, porcine lymphocytes adjusted to a cell concentration of 1 × 10^7^ cells/mL were used as effector cells. The K-562 chronic human myelogenous leukemia cells (American Tissue Type Culture Collection, Manassas, VA, USA) adjusted to a constant 10,000 cells per well were used as target cells. Results were measured using a microplate reader (Bio-Tek, Winooski, VT, USA), and the assay was considered valid if the maximum release divided by spontaneous release was ≤20%.

Mitogen-induced lymphocyte proliferation assay (LPA) was measured using a commercially available non-radioactive cell proliferation assay kit (CellTiter96^®^, Promega, Madison, WI, USA) as previously described by Sutherland et al. [18] with slight modifications to assess adaptive immune status. Briefly, lymphocytes were used at a 5 × 10^6^ cells/mL concentration and placed in a sterile 96-well flat-bottom plate. Concanavalin A (ConA) and lipopolysaccharide (LPS) were used as mitogens (Sigma, St. Louis, MO, USA) to stimulate T and B cells respectively at a concentration of 0, 0.2, 2.0, and 20 μg/mL. Plates were incubated for 68 h at 37 °C in a 5% CO_2_ humidified incubator, and then 15 μL of dye was added and plated incubated for an additional 4 h. The reaction was stopped by adding 100 μL of stop solution to each well. Plates were read using a microplate reader (BioTek Instruments, Winooski, VT, USA) at a wavelength of 550 nm with a reference wavelength of 690 nm. Results are expressed as a proliferation index (PI): Optical Density (550/690 nm) stimulated cells–Optical Density (550/690 nm) non-stimulated cells.

### 2.5. Piglet Body Weight and Litter Processing Practices

Piglet body weights were recorded at birth (0), 1, 7, 14, and 21 (weaning) D-of-age, and then again at 24 h, D7, and D14 post-weaning. Additionally, at 1 D-of-age, piglets were subjected to litter processing procedures, including tail-docking, ear-notching, and an iron injection following standard operating procedures of the research unit at the University of Illinois (Urbana, IL, USA). The pre-processing blood sample was obtained prior to performing any procedures. The post-processing samples were taken after the entire litter was processed and before returning the pigs to farrowing crates. The average time elapsed between the pre-and post-samples was 18 ± 2 min.

### 2.6. Statistical Analysis

Data were analyzed using the mixed procedure of SAS 9.4 with repeated measures (SAS Inst. Inc., Cary, NC, USA). All traits were tested for departure from a normal distribution. The main fixed effects were treatment, age of piglets (birth, 1, 7, 14, 21, 28, and 35), or days post-weaning (0 (weaning), 1, 7, and 14) for the piglets and 2-way interactive effects and random effect was a sow. Fixed effects for sow were treatment and day (24 h post-farrowing and end of lactation). Significance was set at (*p*-value ≤ 0.05), but trends were discussed at (*p*-value > 0.05) to (*p*-value ≤ 0.10).

## 3. Results

### 3.1. Maternal Gestation Treatment on Progeny

#### 3.1.1. Interactive Effect on Cortisol and Immune Profile

**Figure 1:**
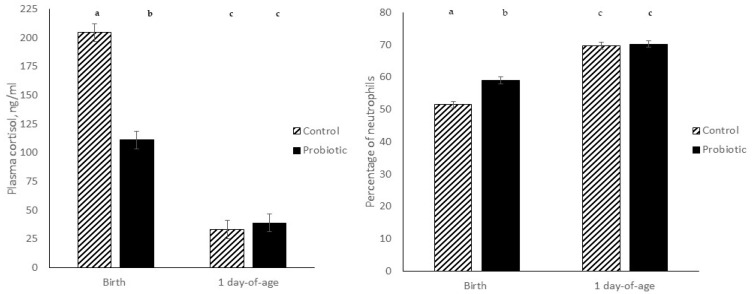
Effect of prenatal maternal treatment on plasma cortisol concentrations (**left**) and percentage of neutrophils (**right**) of piglets at birth and 24-h later. Data are expressed as means ± SEM, and means with different letters differ at *p* < 0.05 (treatment × day of age).

A two-way TRT × Day effect occurred but only for plasma cortisol concentrations and % neutrophils between piglets born to PRO- and CON-fed sows indicating that maternal treatment can affect the progeny in utero (Figure 1). Although in both treatment groups, plasma cortisol concentrations decreased and % neutrophils increased from birth to 24 h later, the magnitude of change of the percent difference was greater in CON piglets. Plasma cortisol concentrations were reduced by 84% and 65% (*p* < 0.0001), and % neutrophils increased by 36% and 19% (*p* < 0.050) in CON and PRO piglets, respectively from birth to 24 h later (TRT × Day; Figure 1). The greater % change in the CON piglets is probably due to the higher cortisol levels and lower % neutrophils at birth. All other immune measures were not significantly different from birth to 24 h later between treatment groups (*p* > 0.110; TRT × Day).

Conversely, there were no maternal TRT effects in response to litter processing procedures (*p* > 0.150; TRT × Time; data not shown). All piglets responded similarly to this stressor regardless of TRT, except for cortisol concentrations (*p* < 0.001). Cortisol concentrations substantially increased from baseline levels, with the percent change being 110% and 86% in PRO and CON piglets, respectively. It is important to note that the overall mean cortisol among PRO piglets was 46% higher than CON, implying they had a more robust cortisol response to this specific stressor.

#### 3.1.2. Treatment Effects at Birth

Overall, maternal feeding of *Scb* to sows during gestation affected cortisol, total WBC counts, and leukocyte DIFF of the progeny before suckling, implying in utero TRT effect (Table 1). At birth, the most profound TRT effects were for cortisol (*p* < 0.0001) and total WBC counts (*p* = 0.006). More specifically, the pigs born to PRO-fed sows, plasma cortisol was 60% lower, and total WBC counts 35% were higher than those born to CON-fed sows. Thus, implying that maternal gestational supplementation of probiotics effectively altered the stress responsiveness of piglets to farrowing stress. Despite the PRO-born piglets having elevated % neutrophils (*p* = 0.004) and reduced % lymphocytes (*p* = 0.002) resulting in higher neutrophil-to-lymphocyte ratio (N:L; *p* = 0.0001), which is often indicative of a greater stress response [19,20]. Conversely, based on lower cortisol and elevated immune cells, including WBC counts and % monocytes (*p* = 0.019), PRO piglets were less likely to be more stressed based on these parameters. Thus, we speculate that perhaps farrowing stress was more stressful to the CON piglets based on these profiles.

#### 3.1.3. Overall Treatment Effect on the Neonate

We also found that maternal gestational treatment may be driving the neonatal effects found at 24 h of age (1 D-of-age) based on significant TRT effects for multiple measures, including mean plasma cortisol (*p* < 0.0001), total WBC counts (*p* = 0.019), % lymphocytes (*p* < 0.005), % neutrophils (*p* = 0.0231), and % monocytes (*p* = 0.002) detected in the progeny born to PRO- and CON-fed sows, with the CON piglets having an overall profile indicative of being more stressed (Table 2). Mean cortisol and % lymphocytes were 37% and 14% higher in CON piglets, whereas mean total WBC counts, % neutrophils, and % monocytes were 21%, 6%, and 38% higher in PRO piglets (Table 2). Surprisingly, mean differential response for these measures among the progeny was reflective of their dams; the only exception was for % neutrophils with PRO-fed sows having lower % than CON-fed (PRO, 33.3 ± 1.1% vs. CON, 44.9 ± 1.2%; *p* < 0.001), which was opposite of their progeny’s neutrophil profile. At 24 h post-farrowing (31 days post-treatment), plasma cortisol concentration was less (PRO, 63.4 ± 7.1 ng/mL vs. CON, 89.5 ± 8.0 ng/mL; *p* < 0.001), and total WBC counts were higher (PRO, 2.5 ± 0.3 10^7^/mL vs. CON, 1.4 ± 0.3 10^7^/mL; *p* < 0.050) among PRO-fed sows. These measures were similar to their progeny, indicating that physiological responses to farrowing stress were similar.

### 3.2. Effects of Maternal Treatment on Piglets during Suckling

Maternal treatment effects during the suckling period on cortisol and descriptive and functional immune measures were also assessed (Table 3). A TRT × Age interactive effect was identified for certain immunological measures. These measures included percentages of lymphocytes and neutrophils, N:L ratio, and LPS-induced lymphocyte proliferation (Table 3). Specifically, at 7 D-of-age, those born to and nursed by PRO-fed sows had fewer % neutrophils and more % lymphocytes resulting in a lower N:L ratio, implying that the PRO piglets may have experienced less stress than control piglets (Table 3). Conversely, it took an additional seven days (14 D-of-age) for these immune parameters to change, resulting in a reduced N:L ratio among the piglets born to CON-fed sows, indicating that at 14 D-of-age, the CON piglets stress response was diminished (Table 3).

Whereas, at 14 and 21 D-of-age the differential treatment effects on functional immune measures including neutrophil chemotaxis (*p* = 0.007), phagocytosis (*p* = 0.036), and ConA-induced (*p* = 0.053) and LPS-induced (*p* = 0.067) lymphocyte proliferation in progeny are reflective of nursing their dams during lactation (Table 3). At 14 D-of-age, neutrophil chemotaxis in response to C5a and IL-8 was significantly different, with PRO piglets having greater chemotaxis than CON. Also, chemotaxis was different at 7 and 14 D-of-age among the PRO piglets. Conversely, at 7 D-of-age, neutrophil phagocytosis was greater among both TRT groups than other days of age, but by 21 D-of-age, phagocytosis was reduced among CON pigs compared to PRO pigs (Table 3). Finally, ConA mitogen-induced T-cell proliferative indexes were similar between PRO and CON pigs at 7 and 14 D-of-age. However, at 21 D-of-age, the CON pigs had a higher index than PRO pigs. Conversely, PRO piglets had a more stimulated LPS mitogen-induced B-cell proliferative index at 7 D-of-age. However, at 14 D-of-age, the index was lower only to return to baseline at 21 D-of-age. All other measures, including cortisol, IL-12, and other immune measures, were not different at 7, 14, or 21 D-of-age (*p* > 0.15; Table 3).

### 3.3. Effects of Maternal Treatment on Pigs to Weaning Stress

Pigs’ responsiveness to weaning stress assessed at 24 h, D7 and D14 post-weaning was differentially affected by maternal treatment (Table 4). A two-way interactive effect of TRT × Day post-weaning occurred for plasma cortisol (*p* = 0.023), total WBC counts (*p* < 0.001), numbers of lymphocytes (*p* = 0.052) and neutrophils (*p* = 0.061), N:L ratio (*p* = 0.012), neutrophil phagocytosis (*p* < 0.008) and chemotaxis (*p* = 0.053), and ConA- (*p* = 0.021) and LPS- (*p* = 0.072) mitogen induced lymphocyte proliferation (Table 4).

Maternal treatment effects were found post-weaning despite only a few differences in immune measures before weaning. Specifically, at D1 post-weaning, plasma cortisol remained elevated in PRO pigs but decreased among the CON pigs by 17.6% and continued to decrease, resulting in cortisol concentrations being 52.6% lower by D14 post-weaning in CON pigs (Table 4). Conversely, it took D7 post-weaning before cortisol concentration decreased by 42% compared to levels at D1 post-weaning in PRO pigs (*p* < 0.001), and by days 7 and 14, cortisol concentrations were similar between PRO and CON pigs (Table 4). Moreover, at D1 post-weaning, total WBC counts were reduced only in PRO pigs, but lymphocyte counts were reduced in PRO and CON pigs. Conversely, it took D14 post-weaning before there were differences in neutrophil numbers between PRO and CON pigs; neutrophil numbers were elevated by 93.7% and 86.1% in CON and PRO pigs, respectively at D14 post-weaning compared to before weaning. Still, CON pigs had more neutrophils than PRO pigs, with some of these effects being detected at birth and during lactation on cortisol, neutrophils, and N:L ratios.

Although no TRT × Day effect occurred for leukocyte DIFF (*p* > 0.116), the N:L ratio increased in PRO, and CON pigs at D1 post-weaning, indicating weaning stress evoked a stress response decreased at D7 post-weaning. However, N:L ratio increased again at D14 post-weaning, but PRO pigs had a significantly lower ratio than CON pigs (Table 4).

A TRT × Day post-weaning effect occurred for neutrophil phagocytosis, chemotaxis in response to IL-8, and mitogen-induced lymphocyte proliferation (Table 4).

At D1 (24 h) post-weaning, neutrophil chemotaxis, and Con-A induced lymphocyte proliferation were enhanced while neutrophil phagocytosis remained unaffected among PRO piglets. Interestingly, at D1 post-weaning, ConA-induced lymphocyte proliferation increased by 32.6% in PRO pigs from D0. Conversely, the ConA index decreased by 30.4% in CON pigs, and at D14 post-weaning neutrophil chemotaxis in response to IL-8 was greatly enhanced among PRO pigs. It should be noted that CON pigs had a greater CON-A index before weaning. Conversely, LPS-induced lymphocyte proliferation was reduced by 24.1% at D7 post-weaning among CON pigs, while at D14 post-weaning, the LPS index was increased by 78.5% among PRO pigs compared to D0 (Table 4). No TRT × Day post-weaning effects were found for natural killer cell cytotoxicity or plasma IL-12 (Table 4; *p* > 0.20).

### 3.4. Effects of Maternal Treatment on Body Weight

**Figure 2:**
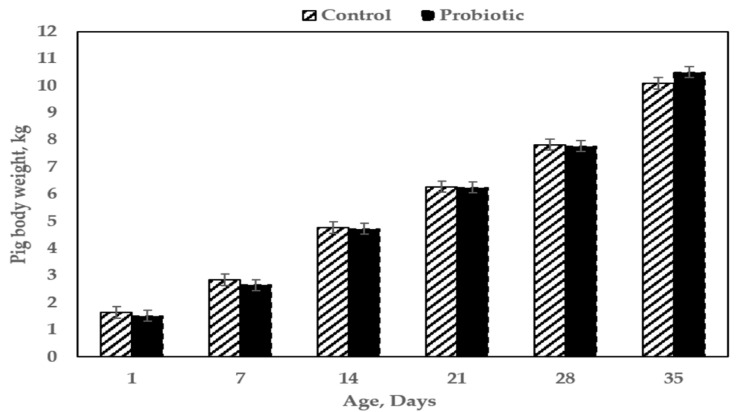
Body weights for pigs born to sows individually fed either control (sugar-based placebo) or probiotic (*Saccharomyces cerevisiae* var. *boulardii*) boluses from late-gestation through the end of lactation.

Feeding sows *Scb* from late-gestation until farrowing (31 D of treatment) and continuation of feeding *Scb* throughout lactation did not affect pig body weight from 1 D-of-age through 35 D-of-age (*p* = 0.764; Figure 2).

## 4. Discussion

Maternal stress and its impact on the offspring can occur both pre-and postnatal, affecting the health and stress response of the progeny. Prenatal stress exposes the progeny to elevated circulating cortisol levels in utero [21]. The transfer of glucocorticoids (i.e., cortisol) from mother to fetus plays an important role in fetal programming of offspring’s hypothalamic-pituitary-adrenal (HPA) axis. The transfer occurs via direct effects on the brain or crossing the placenta and downregulating and affecting brain development [22]. Offspring from experimentally stressed sows show an exaggerated HPA axis in response to common stressors [23], partly explained by glucocorticoid surge altering gene regulation in organs and tissues of the offspring. Pigs born to and nursed by sows fed *Saccharomyces cerevisiae boulardii* during late-gestation and lactation had unique cortisol/immune profiles due to farrowing and weaning stress. Thus, implying maternal *Scb* supplementation can affect her progeny’s immune and stress responses both prenatally and postnatally. Moreover, the sows’ biological response may have stimulated her progenys’ immune system or altered cortisol to immunoenhancing levels to prepare the immune system for surveillance and protection [1]. Overall, pigs born to *Scb*-fed sows had a less robust cortisol response to farrowing stress but more robust to litter processing and weaning stressors. At the same time, the immune status in response to these stressors was often more stimulated, especially in terms of neutrophil function and B-cell lymphocyte proliferation, and appeared to return to non-stress levels faster than control pigs.

Prenatal stress during late-gestation can impair the development and reactivity of the immune system of the progeny and impact the frequency of disease and mortality [4]. Thus, diminishing the negative effects of early life stressors and enhancing the immune response would be beneficial, especially in the first weeks of life. However, Collier et al. [13] showed that the cumulative Scb-induced immune-neuroendocrine LPS response of delayed cortisol and oxidative burst might function to facilitate short-term pathogen clearance. Factors such as environmental stressors, husbandry practices, and antigenic exposures of sows may negatively affect the early development of their progeny’s immune system [4,23,24,25,26,27]. Since blood samples were taken prior to nursing, these data support that it is feasible to modulate the stress response of the progeny in utero, even late in gestation. The significantly lower cortisol levels and more stimulated immune cell profiles found at birth and 24-later among the pigs born to the *Scb*-fed sows indicated that the progeny’s stress responsiveness to a farrowing stressor is due to prenatal treatment. The progeny’s cortisol profile was reflective of their dam’s response 24-h post-farrowing. Previous research has indicated that plasma cortisol secretion in sows increases at farrowing and weaning [28,29]. Plasma cortisol did increase to levels indicative of stress in both treatment groups at farrowing; however, the *Scb*-fed sows and progeny cortisol response at farrowing and 24-h later was diminished compared to the controls. These results may also reflect effects occurring in utero in terms of reduced cortisol at the maternal-fetal interface at the onset of parturition. Surprisingly, there was no prenatal treatment effect in response to litter processing stressors, including handling, ear notching, and tail-docking. Both treatment groups perceived it as a stressful event, as evident by the substantial increase in cortisol concentrations following these procedures, but there were no adverse effects on other measures.

Although it is difficult to delineate between prenatal and postnatal treatment effects on the progeny, especially during lactation, these findings confirm that maternal influence does not solely occur in the postnatal period. There is evidence that an in utero exposure via sow’s intake of *Scb* and possibly her physiological response to the probiotic and stress of pregnancy and farrowing altered the cortisol response and several immune parameters compared to control animals. It is also possible that there were prenatal immune-modulatory carry-over effects up to 7 days-of-age since the PRO pigs had a profile indicative of a less stressed animal compared to the control pigs. They had a more stimulated innate (neutrophil chemotaxis) and adaptive (LPS-induced lymphocyte proliferation) immune system. In contrast, the control animals did not achieve homeostasis until 14 days-of-age evident by reduced N:L ratio indicating a less stressed animal. However, since the progeny did not receive *Scb* directly, the effects beyond and through weaning are due to exposure to the *Scb* product predominantly via sow’s milk. We did not find effects of *Scb* on body weight from birth through 14 days post-weaning; only immune function was stimulated. Chevaux and Guillou [30] found that *Scb* supplemented in sow diets during peripartum and farrowing periods improved the quality of colostrum and milk, directly affecting the well-being of pigs during nursing. Jurgens et al. observed that the inclusion of *Saccharomyces cerevisiae* CNCM I-4407, a strain of *S. cerevisiae* closely related to the probiotic fed here, increased antibody concentration in both colostrum and milk, resulting in enhanced piglet immunity in the postnatal period [31]. The inclusion of *Saccharomyces cerevisiae* var. *boulardii* improved milk quality via an increased fatty acid profile [32]. Since we did not collect milk samples nor feed *Scb* as long as they did, we do not know if milk quality was altered by *Scb* treatment.

Moreover, the progeny’s unique cortisol/immune profile to weaning stress may not only be due to nursing but also coprophagy or a combination of sources. Brousseau et al. reported that offspring from probiotic-treated dams had high amounts of live *Pediococcus acidilactici* and *Saccharomyces cerevisiae* subsp. *boulardii* in feces and intestinal content [11]. Thus, indicating that ingested probiotics were efficiently delivered in the gut of piglets during lactation and after weaning. In that same accord, the presence of both live yeast products in offspring feces may elude that *Scb* may be present in the dam’s feces. Without removing sows’ feces, offspring may be directly exposed to the probiotic through coprophagy or other contact paths. Nevertheless, it is plausible that the piglet immune system and stress responsiveness before and after weaning were further impacted by nursing the *Scb*-fed sows during lactation or coming in contact with feces from the sows.

Finally, during the weaning transition, pigs have to cope with unexpected abrupt separation from the sow and move to a new environment and dietary changes, often associated with a high occurrence of post-weaning diarrhea, which can trigger potential enteric pathogens [33]. However, limited data have explored the effects on immune and stress responsiveness of pigs born to and nursed by *Scb*-fed sows. The PRO-born and nursed pigs continued to show an increased innate immune response through significantly higher neutrophil % and rates of chemotaxis in response to both chemoattractants and enhanced B-cell induced proliferation through the lactation period. Contrastingly, it was found that when piglets were directly fed *Saccharomyces cerevisiae,* they displayed higher lymphocyte percentages and lower neutrophil percentages than pigs fed a control [34]. These differences may be due to directly feeding yeast to the pigs compared to our data supporting prenatal and postnatal maternal effects of *Scb* on immune measures and activation of the HPA axis via cortisol. There are reports that feeding yeast cultures to pigs change the gut’s immune function by triggering the T-helper 1 (Th-1) response due to increased cytokine IFN-γ [35]. While in another study conducted by Gao et al. in broiler chickens, they found that feeding yeast cultures increased the lysozyme in the gut, indicating an increased nonspecific immune function [36]. Still, others found that neutrophils were highest among control pigs. Contrarily, those fed probiotics had reduced neutrophils and increased immunoglobulin G [37]. These data imply that probiotics may enhance offspring’s innate and adaptive immune response from probiotic-treated dams, reducing these piglets’ stress responsiveness to multiple stressors. Shen et al. fed gestating sows *Saccharomyces cerevisiae* fermented for the entire gestation and lactation phases found no effects on progeny at birth or 17 days-of-age for any immune measures assessed [38]. Others have shown that probiotics can act against infection and induce IL-12 production, activating NK cells and developing Th-1 cells [39]. However, we did not find any treatment effect on NK or IL-12 levels in response to the stressors used in this study; these measures were affected by piglet age (data not shown). Despite not finding treatment effects on NK cytotoxicity within, we did detect treatment differences on neutrophil function in response to weaning stress.

The innate immune system consists of nonspecific defense mechanisms and cells such as neutrophils which are phagocytic [40]. Early on, it seems like probiotics impacted innate immune parameters, exerting an immediate or near-immediate response to the presence of pathogens in the body as evidenced by a higher neutrophil % and N:L at birth. On the other hand, adaptive immunity is highly specific and can destroy individual invading pathogens [41]. It is possible that we were starting to detect some long-lasting protective memory responses in the probiotic pigs in the sense of a more robust B cell response. Published articles regarding the immune responses of probiotics are few in numbers, especially when assessing maternal effects of feeding probiotics on the progeny in the short and long term. Therefore, the supplementation of the yeast probiotic boluses, *Scb*, during the gestation period to weaning did impact the immune status and stress response of PRO-sows during gestation and lactation. Feeding *Scb* to PRO-sows during gestation and lactation may have had a maternal-fetal interaction, thereby transferring the dam’s immune status and stress responsiveness effects to her offspring. The use of probiotic-supplemented feeds may lessen the impact of animal health issues and other unavoidable stressors that often challenge food animals.

## 5. Conclusions

Overall, feeding sows yeast probiotic boluses of *Scb* during late-gestation through lactation resulted in positive outcomes on innate and adaptive immune responses in their progeny in response to farrowing and weaning stressors. The pigs born to and nursed by *Scb*-fed sows had unique cortisol/immune profiles in response to farrowing and weaning stressors. The PRO pigs had a less robust stress response; others had a more robust response without negative consequences. In contrast, others were more robust stress responses without negative consequences. These pigs often had more stimulated immune function following stress or returned to a non-stressed state faster than controls. These data imply that we may use probiotic supplementation to lessen the impact of animal health issues and other unavoidable stressors that often challenge food animals. However, future research needs to evaluate different time courses, strains, and doses to have longer and more influential effects on pigs in response to stress. Moreover, future research may also consider directly feeding probiotics to pigs, particularly around weaning, to potentially reduce the negative impact of stressors beyond the 14 days post-weaning.

## Figures and Tables

**Table 1:** Prenatal maternal gestational treatment effects on the progeny mean plasma cortisol, total white blood cell counts, leukocyte differentials, neutrophil-to-lymphocyte ratio, and interleukin-12 at birth (prior to suckling) ^1,2^.

Measures	Control	Probiotic	SEM	*p*-Value
Plasma cortisol, ng/mL	205.8	111.1	9.21	<0.000
White blood cell, 10^7^/10 µL	1.67	2.37	0.180	0.006
Lymphocytes, %	47.1	38.9	1.09	0.002
Neutrophils, %	51.5	59.0	1.17	0.004
Monocytes, %	0.98	1.57	0.180	0.019
Eosinophils, %	0.44	0.29	0.073	0.149
Neutrophil-to-lymphocyte ratio	1.16	1.84	0.122	<0.001
Interleukin-12 pg/mL	172.4	170.0	9.15	0.736

^1^ Data are shown as least squares means ± standard error of mean (SEM) (n = 42 piglets per sow treatment). ^2^ Gestational treatments were control = sugar-based placebo and probiotics = *Saccharomyces cerevisiae* var. *boulardii.*

**Table 2:** Prenatal maternal gestational treatment effects on the progeny mean plasma cortisol, white blood cell counts, leukocyte differentials, neutrophil-to-lymphocyte ratio, and interleukin-12 at 24 h post-birth ^1,2^.

Measures	Control	Probiotic	SEM	*p*-Value
Plasma cortisol, ng/mL	119.0	75.1	6.20	<0.000
White Blood Cell, 10^7^/10µL	2.03	2.51	0.142	0.019
Lymphocytes, %	37.6	32.2	1.20	<0.005
Neutrophils, %	60.6	64.6	1.02	0.023
Monocytes, %	1.31	2.11	0.182	0.002
Eosinophils, %	0.46	0.51	0.078	0.626
Neutrophil-to-lymphocyte ratio	2.34	2.62	0.285	0.507
Interluekin-12, pg/mL	183.5	190.1	7.49	0.531

^1^ Data are shown as least-square means ± standard error means (SEM) represents n = 42 piglets per sow treatment. ^2^ Gestational treatments were controls = sugar-based placebo and probiotics = *Saccharomyces cerevisiae* var. *boulardii*.

**Table 3:** Interactive effects of maternal treatment on the progeny’s mean plasma cortisol and descriptive and functional immune measures during the suckling phase at 7, 14, and 21 days of age (before weaning) ^1,2^.

	7D		14D		21D			
Measures	Control	Probiotic	Control	Probiotic	Control	Probiotic	SEM	Treatment × Age
Plasma cortisol, ng/mL	43.4	42.4	37.1	45.6	44.2	50.8	6.00	0.493
White blood cell, 10^7^/10 µL	6.29	6.10	3.49	3.34	1.41	2.24	0.70	0.697
Lymphocyte, 10^7^/mL	3.96 ^a^	4.86 ^a^	3.70 ^a^	4.68 ^a^	8.94 ^b^	4.03 ^a^	2.01	0.005
Neutrophil, 10^6^/mL	4.43	3.86	2.49	2.71	2.64	2.04	0.53	0.432
Lymphocytes, %	43.9 ^a^	53.0 ^b^	60.4 ^c^	64.4 ^cd^	65.7 ^d^	65.4 ^cd^	2.23	0.060
Neutrophils, %	53.7 ^a^	44.4 ^b^	37.4 ^c^	32.7 ^c^	33.0 ^c^	32.5 ^c^	2.25	0.088
Monocytes, %	2.47 ^a^	2.20 ^ab^	1.88 ^a^	1.17 ^ad^	1.69 ^ac^	2.13 ^ab^	0.43	0.165
Eosinophils, %	0.27	0.40	0.46	0.53	0.42	0.36	0.13	0.758
Neutrophil-to-lymphocyte ratio	1.62 ^a^	0.97 ^b^	0.69 ^c^	0.61 ^c^	0.57 ^c^	0.61 ^c^	0.11	0.001
Chemotaxis-C5a, no./5 fields	31.2 ^a^	31.6 ^a^	39.9 ^a^	68.2 ^b^	110.2 ^c^	115.4 ^c^	4.94	0.052
Chemotaxis- IL8, no./5 fields	34.9 ^a^	32.1 ^a^	56.3 ^b^	84.6 ^c^	125.1 ^d^	92.4 ^c^	6.21	0.007
Neutrophil phagocytosis, %	67.4 ^ab^	68.7 ^a^	63.4 ^ab^	61.1 ^bc^	56.0 ^c^	62.2 ^b^	3.09	0.036
Natural killer cell cytotoxicity, %	54.9	53.4	58.6	66.9	49.7	67.7	7.81	0.605
Concanavalin-A proliferation	1.02 ^a^	1.25 ^ab^	1.18 ^ab^	1.27 ^ab^	2.47 ^c^	1.75 ^d^	0.12	0.053
Lipopolysaccharide proliferation	1.39 ^a^	1.76 ^b^	1.31 ^a^	1.14 ^c^	1.46 ^a^	1.72 ^b^	0.20	0.067
Interleukin-12, pg/mL	165.6	158.1	259.7	276.8	296.3	317.6	17.95	0.425

^1^ Data are shown as least-squares mean ± pooled standard error mean (SEM) represents n = 42 piglets per sow treatment. ^a–d^ Within a row, means without a common superscript letter differ at *p* ≤ 0.05, the *p*-value is the interactive effect of sow treatment × piglet age (Treatment × Age). ^2^ Sow treatments were controls = sugar-based placebo and probiotics = *Saccharomyces cerevisiae* var. *boulardii.* 7D = 7 days-of-age; 14D = 14 days-of-age; 21D = 21 days-of-age.

**Table 4:** Interactive effects of maternal treatment by days post-weaning on pig plasma cortisol and descriptive and functional immune measures in response to weaning stress ^1,2^.

	D0		D1		D7		D14			
Measures	Control	Probiotic	Control	Probiotic	Control	Probiotic	Control	Probiotic	SEMp	Treatment × Day
Plasma cortisol, ng/mL	44.4 ^a^	50.8 ^a^	36.4 ^b^	53.3 ^a^	24.7 ^c^	31.1 ^bc^	25.8 ^c^	25.1 ^c^	4.00	0.023
White Blood Cell (WBC), 10^8^/mL	1.41 ^a^	2.21 ^a^	2.19 ^a^	1.35 ^b^	2.30 ^b^	2.08 ^ab^	3.37 ^c^	3.51 ^c^	0.28	<0.001
Lymphocyte, 10^7^/mL	8.94 ^a^	4.03 ^b^	3.44 ^bc^	1.58 ^c^	3.80 ^b^	2.51 ^bc^	5.25 ^b^	3.72 ^b^	1.31	0.052
Neutrophil, 10^6^/mL	2.64 ^a^	2.04 ^a^	1.76 ^a^	1.64 ^a^	2.26 ^a^	2.02 ^a^	7.29 ^b^	5.13 ^c^	0.47	0.061
Lymphocytes, %	65.7	65.2	56.1	57.5	59.0	61.0	47.7	56.3	2.40	0.143
Neutrophils, %	33.0	32.1	41.1	39.6	37.1	33.9	49.7	40.9	2.51	0.196
Monocytes, %	1.69	2.13	2.54	2.44	3.04	3.92	1.71	1.87	0.59	0.504
Eosinophils, %	0.41	0.35	0.38	0.30	0.52	1.04	0.96	0.87	0.15	0.116
Neutrophil-to-lymphocyte ratio	0.57 ^a^	0.60 ^a^	0.85 ^b^	0.87 ^b^	0.72 ^ab^	0.63 ^a^	1.23 ^c^	0.81 ^b^	0.10	0.012
Chemotaxis-C5a, no.	110.2	115.4	60.1	64.5	75.3	58.3	67.8	64.1	13.2	0.858
Chemotaxis-IL-8, no.	125.1 ^a^	92.4 ^b^	118.8 ^a^	143.8 ^c^	65.0 ^d^	66.7 ^d^	113.4 ^a^	173.1 ^e^	9.3	0. 053
Neutrophil phagocytosis, %	56.0 ^a^	62.3 ^b^	70.1 ^c^	65.3 ^bc^	60.5 ^b^	62.3 ^b^	66.2 ^c^	68.3 ^c^	1.91	<0.008
Natural killer cell cytotoxicity, %	49.7	67.7	33.2	35.7	70.7	71.5	47.7	45.0	5.75	0.237
ConcanavalinA proliferation	2.47 ^a^	1.75 ^b^	1.72 ^b^	2.32 ^a^	2.36 ^a^	2.43 ^a^	2.18 ^ab^	2.06 ^ab^	0.23	0.021
Lipopolysaccharide proliferation	1.45 ^ab^	1.72 ^a^	1.65 ^a^	1.89 ^a^	1.10 ^b^	1.63 ^a^	1.65 ^a^	3.07 ^c^	0.38	0.072
Interluekin-12, pg/mL	296.3	317.4	230.0	258.7	419.3	411.8	312.9	353.5	23.7	0.312

^1^ Data are shown as least-squares means ± pooled standard error means (SEMp) represents n = 42 piglets per sow treatment. ^a–e^ Within a row, means with different superscript differ at *p* < 0.05, *p*-value is the interactive effect of sow treatment × day post-wean (Treatment × Day). ^2^ Sow treatments were controls = sugar-based placebo and probiotics = *Saccharomyces cerevisiae* var. *boulardii.* Days (D) post-weaning are represented as 0D = prior to weaning, 1D = 24 h post-wean, 7D = 7 days post-wean, and 14D = 14 days post-wean.

## Data Availability

Data presented in this study are available upon request from the corresponding author.
